# Data-driven patient stratification of UK Biobank cohort suggests five endotypes of multimorbidity

**DOI:** 10.1093/bib/bbac410

**Published:** 2022-10-08

**Authors:** Bodhayan Prasad, Anthony J Bjourson, Priyank Shukla

**Affiliations:** Personalised Medicine Centre, School of Medicine, Ulster University, UK. He holds a MSc in Computational and Integrative Sciences from Jawaharlal Nehru University, India; Personalised Medicine Centre, School of Medicine, Ulster University, UK. He holds a PhD in Genomics and Molecular Biology from Queen's University, Northern Ireland; Personalised Medicine Centre, School of Medicine, Ulster University, UK. He holds a PhD in Computer Science with area of research in Bioinformatics from University of Bologna, Italy

**Keywords:** multimorbidity, comorbidity, UK Biobank, multiple correspondence analysis (MCA), charlson comorbidity index (CCI), clustering, patient stratification, endotypes, machine learning, disease interaction network

## Abstract

Multimorbidity generally refers to concurrent occurrence of multiple chronic conditions. These patients are inherently at high risk and often lead a poor quality of life due to delayed treatments. With the emergence of personalized medicine and stratified healthcare, there is a need to stratify patients right at the primary care setting. Here we developed multimorbidity analysis pipeline (MulMorPip), which can stratify patients into multimorbid subgroups or endotypes based on their lifetime disease diagnosis and characterize them based on demographic features and underlying disease–disease interaction networks. By implementing MulMorPip on UK Biobank cohort, we report five distinct molecular subclasses or endotypes of multimorbidity. For each patient, we calculated the existence of broad disease classes defined by Charlson's comorbidity classification using the International Classification of Diseases-10 encoding. We then applied multiple correspondence analysis in 77 524 patients from UK Biobank, who had multimorbidity of more than one disease, which resulted in five multimorbid clusters. We further validated these clusters using machine learning and were able to classify 20% model-blind test set patients with an accuracy of 97% and an average Jaccard similarity of 84%. This was followed by demographic characterization and development of interlinking disease network for each cluster to understand disease–disease interactions. Our identified five endotypes of multimorbidity draw attention to dementia, stroke and paralysis as important drivers of multimorbidity stratification. Inclusion of such patient stratification at the primary care setting can help general practitioners to better observe patients’ multiple chronic conditions, their risk stratification and personalization of treatment strategies.

## Introduction

Multimorbidity generally refers to the occurrence of more than one chronic disease [1]. Chronic diseases are those that are persistent and long-lasting and include arthritis, diabetes and high blood pressure amongst many others. These chronic conditions can be physical non-communicable diseases of long duration such as cardiovascular disease or cancer, a mental health condition of long duration such as a mood disorder or dementia or an infectious disease of long duration such as HIV or hepatitis C [2]. For example, the study by Pieringer and Pitchler in 2011 [3] and the Center for Disease Control and Prevention report [4] on patients with arthritis has reported that 24% suffered from cardiovascular diseases, 19% respiratory conditions, 16% diabetes and 24% depression.

Older population, i.e. people over the age of 40, are more likely to develop multiple chronic conditions (multimorbidity). Hospitals in the UK see around 40–50% older patients [5]. Total long-term care expenditure in 2017 was £48.2 billion (~2% UK GDP), of which approximately two-thirds (66%) was financed by the government and 31% by people who directly paid for the services and medication [6], thus making multimorbidity an economic challenge as well.

With the progress of medical science, patients’ longevity has increased. Global life expectancy now sits around age 72 – more than double that of 100 years ago [7]. But the increased longevity has led to the rise of multimorbidity in patients [8]. In 1900, top three causes of death were infectious diseases like pneumonia and flu, tuberculosis, and gastrointestinal infection [9]. By 2010, these were replaced by cancer, heart disease and cerebrovascular disease [10]. Further, the mortality from all causes has declined by 54% from 1900 to 2010 [11]. With the advent of modern medicine, life expectancy has been gradually increasing. The data for 2018–20 show that the life expectancy at birth for UK has now reached 79 years for males and 82.9 years for females [12]. The difference between the genders is also gradually decreasing as the male life expectancy is increasing at a faster rate than females [13]. Additionally, healthy life expectancy data show that it is 62.9 years for males and 63.3 years for females for 2017–19 [14]. The difference between the life expectancy and healthy life expectancy is years a patient spends in poor health. This difference is about 19.1 years (64 years in good health) for 2012–14 and was 18.1 years (62.5 years in good health) in 2000–02 [15]. For both the sexes, years in poor health from age 65 has increased by 1.4 years for females and 1.5 years for males in 2012–14 as compared to 2000–02 [15]. Hence, multimorbidity is not just a special case, but a norm in today’s world.

There exist many metrics to measure multimorbidity and/or comorbidity beyond a simple disease count [16]. One of the first study in this field by Charlson *et al.* in 1987 [17] suggested Charlson Comorbidity Index (CCI) as a weighted metric for multimorbidity, giving weights to 17 broad disease classes based on severity that can decrease longevity. Thus, a higher CCI means the patient is more multimorbid and hence prone to die early. The CCI was later standardized using the International Classification of Diseases-10 (ICD-10) by Quan *et al*. in 2005 [18]. The CCI is the most widely used multimorbidity measurement metric [16]. Therefore, for the current scope of our study we have used it for describing multimorbidity in UK Biobank cohort and have focused on the patients having at least two disease classes defined by the Charlson’s comorbidity classification.

Comorbidity is often used interchangeably with multimorbidity, but there is a subtle difference between the two. Traditionally, a patient with multimorbidity visits specialists for each disease in secondary care settings. These patients are labelled with one disease as the major disease *alias* index disease or condition and the rest of the conditions are labelled as comorbidities. This approach makes the specialist treat and mostly consider the major condition. In contrast, multimorbidity is where multiple chronic conditions are studied together with their interactions with each other and analyzed under a single umbrella, like how generalist practice in a primary care setting. Thus, index-comorbidity regime deals majorly with one index disease, whereas multimorbidity looks at multiple chronic conditions together along with their interactions with each other [19]. There is a need to renew the relationship between specialists and generalists, who have different but complimentary skills to personalize the treatment of patients with multimorbidity.

At public healthcare systems, such as the National Health Service (NHS) in the UK, patients often enter a long waitlist [20]. There is a current need to explore more sophisticated and stratified or personalized treatment approach that can prioritize treatments for the high-risk patients, especially those with multimorbidity. This has led to stratified or personalized medicine becoming one of the priority research areas for Innovate UK, the Medical Research Council (MRC) UK and the Academy of Medical Sciences UK [21]. The need for a personalized medicine approach in this regard can be achieved by clustering patients using unsupervised machine learning (ML) techniques and then characterizing them using various demographic and clinical data. Here we propose an analytical approach (MulMorPip) based on multiple correspondence analysis (MCA), which is a multivariate technique within unsupervised ML field. MulMorPip is a step towards equipping the clinicians with patient stratification based on multimorbidity and understanding disease–disease interactions within multimorbid groups, which can eventually help them in better decision-making, prioritizing and personalizing the treatment plans for multimorbid patients.

## Material and methods

**Figure 1:**
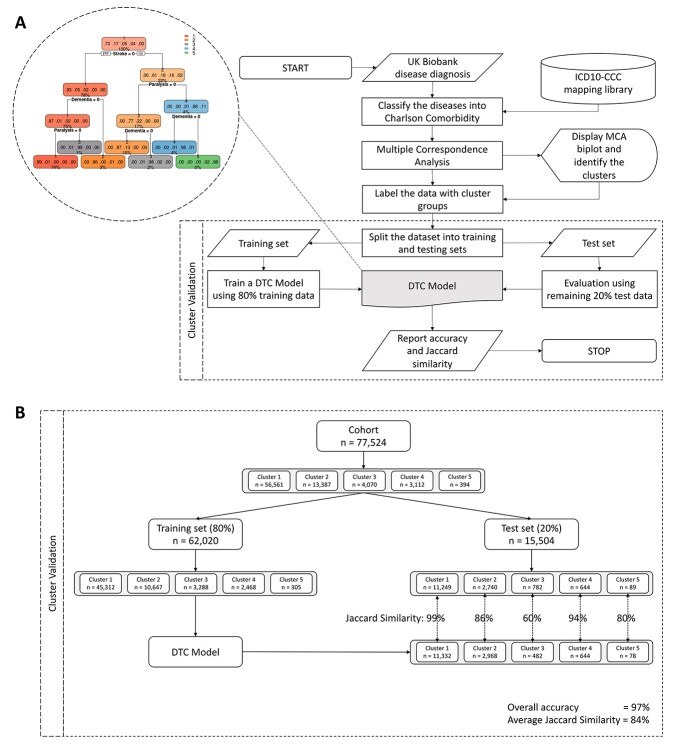
(**A**) Flowchart presentation of multimorbidity analysis pipeline (MulMorPip). Oval shapes represent start/stop, parallelogram boxes represent input/output, rectangular boxes represent computation process and cylinder represents library/database. DTC model has been zoomed-out and presented in the dotted circle. (**B**) Detailed presentation of cluster validation. ICD10 = International Classification of Diseases 10^th^ Revision, CCC = Charlson’s comorbidity classification, DTC = decision tree classifier, MCA = multiple correspondence analysis.

A summary flowchart of the bioinformatics analysis pipeline, namely multimorbidity analysis pipeline (MulMorPip), is presented in Figure 1, and all the code of the pipeline has been made available in the following public repository https://github.com/ShuklaLab/MulMorPip.

### Datasets

UK Biobank (https://www.ukbiobank.ac.uk/) has recruited about 500 000 patients from Great Britain (England, Scotland and Wales). These participants gave consent for access to their electronic care record. We obtained the UK Biobank data via Application No. 48433. We collected the ICD-10 disease diagnosis of the patients from the UK Biobank field id 41270 (https://biobank.ndph.ox.ac.uk/showcase/field.cgi?id=41270). Dataset belonging to participants who later revoked their consent was excluded.

### Statistical, computational and bioinformatic analyses

All statistical and computational analyses were carried out in R v3.6.1 [22]. The t-test and chi-square test to check for demographic variables were performed in the ‘*base*’ package. The UK Biobank data was loaded with the help of ‘*ukbtools*’ package [23] and transformed into subsequent Charlson’s broad disease classes using the ‘*icd*’ package [24]. MCA was carried out using the ‘*FactoMineR*’ package [25] and plotted using the ‘*factoextra*’ package [26]. Data splitting was done using the ‘*caret*’ package [27]. ML model for decision tree was made using ‘*rpart*’ package [28] and plotted using ‘*rpart.plot*’ package [29]. Network analysis was performed using Cytoscape software [30].

### Multiple correspondence analysis

The ICD-10 summary diagnoses of the UK Biobank patients were grouped into Charlson’ broad disease classification. The patients with multimorbidity were obtained using disease count of greater than 1 for the Charlson’s broad disease classification. This subset of patients was then used for performing MCA. The MCA plot was then rotated using matrix multiplication M = [1 1.8; 1.8 –1] to make clusters vertical, which were then partitioned using the x-axis (cut-offs: 0, 0.7, 1.4 and 2.1) and labelled with cluster numbers.

### Cluster validation

We divided the data into training set (80%) and test set (20%), trained a decision tree classifier (DTC) with the training set and used the DTC model to predict the test set. We compared the predictions with the original cluster values using overall accuracy and Jaccard similarity scores. Finally, we plotted the predictions as well as original cluster values in a separate MCA plot of 20% test set. Random number generation seed was set to 200, prior to carrying out validation.

### Network analysis

The prevalence of each disease was calculated for each of the identified clusters and size of the network disease node was then made proportional to it. Further, the co-occurrence of two diseases was obtained and used in defining the thickness of the network edges. Disease interaction networks were then plotted in circular topology.

## Results

### Cluster analysis and its validation

**Figure 2:**
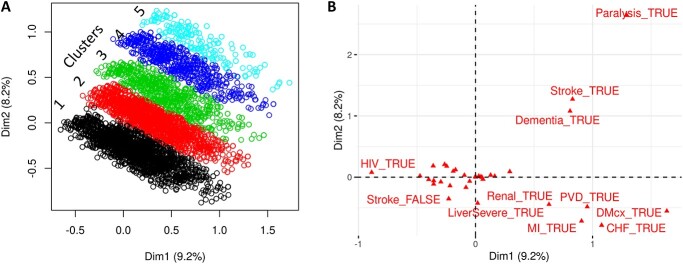
Multimorbid clusters: multiple component analysis (MCA) for Charlson's comorbidity classification of 77 524 patients with multimorbidity in UK Biobank. (**A**) MCA plot showing five different clusters. (**B**) Coordinates of variable categories in the two principal dimensions of MCA plot. Variance explained by MCA dimensions are mentioned under parenthesis. Variables far from *x*,*y* = 0,0 have been labelled. MI = myocardial infraction, CHF = congestive heart failure, PVD = peripheral vascular disease, DMcx = DM with chronic complications, TRUE = disease present, FALSE = disease absent.

We selected the UK Biobank participants belonging to two or more broad disease classes as per Charlson’s classification of multimorbidity. The count of broad disease classes in this multimorbid cohort (n = 77 524) varied from 2 to 13 (Supplementary Table 1). This classification was used to carry out MCA, which showed five distinct clusters (Figure 2A). Plot of variable categories (i.e. 17 broad disease classes defined by Charlson) against principal dimensions showed dependence of clusters on paralysis, followed by stroke and dementia (Figure 2B). In order to validate these clusters, we set aside 20% test data and confirmed that the basic demographic features are representative of the 80% training set (Supplementary Table 2). We then trained a DTC model on the remaining 80% data. The flowchart for the validation scheme is presented in Figure 1A (lower panel), and the corresponding sample sizes and results are shown in Figure 1B. We chose DTC because the disease data is categorical, and DTC is extensively used with categorical data and gives a simple and meaningful decision tree for decision-making. The DTC model obtained using the 80% training set is presented in Figure 1A (zoomed dotted circle). The DTC was seen to use the same three disease classes (i.e. paralysis, stroke and dementia) to define its branching. The performance of the DTC model in terms of confusion matrix is presented in Supplementary Table 3. The prediction of 20% test set using the DTC model gave an overall accuracy of 97% (Figure 1B). However, since the number of patients in each cluster was significantly different, we computed Jaccard similarity for each cluster and obtained an average Jaccard similarity score of 84% (Figure 1B). To visually compare the original clustering results with the validation results, we further did MCA on 20% test set and coloured each data point (patients) using their original cluster membership as well as predicted cluster membership, which showed a huge overlap between the original clustering results with the validation results (Supplementary Figure 1). The overall high prediction accuracy of 97%, average Jaccard similarity score of 84% and MCA plot of 20% test set validate the existence of five multimorbid clusters in the UK biobank cohort.

### Exploratory data analysis on patients with multimorbidity

**Figure 3:**
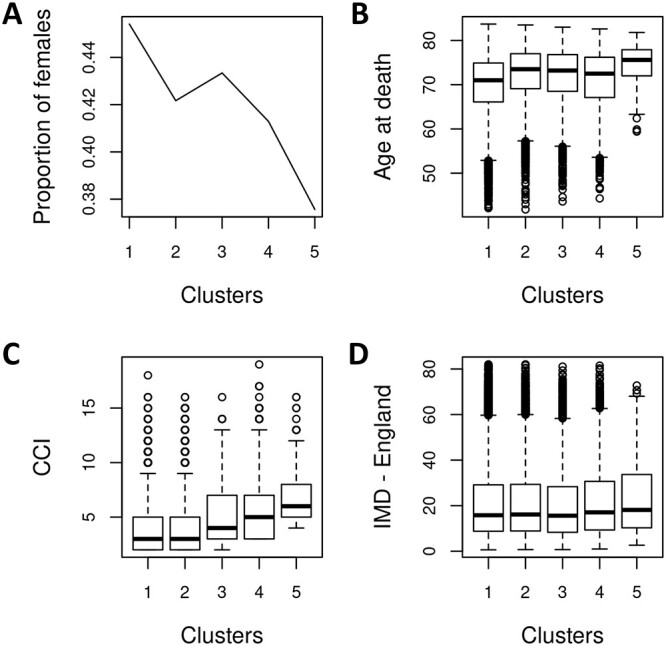
Characterization of clusters based on demographic data. (**A**) Plot of gender distribution shows a decreasing proportion of females from cluster 1 to 5. Box plots of (**B**) age at death, (**C**) Charlson Comorbidity Index (CCI) and (**D**) Indices of Multiple Deprivation (IMD) show significant differences between clusters. IMD classification of England was used.

**Figure 4:**
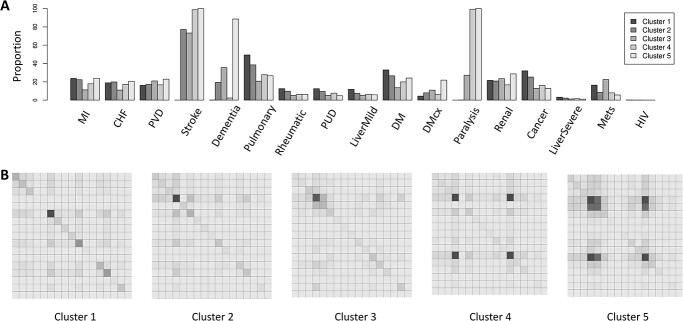
(**A**) Prevalence of 17 broad disease classes as per Charlson’s comorbidity classification. (**B**) Heatmap showing the co-occurrence of 17 broad disease classes as per Charlson’s comorbidity classification. Order of disease from top to bottom and left to right are myocardial infraction (MI), congestive heart failure (CHF), peripheral vascular disease (PVD), stroke, dementia, pulmonary disease, rheumatic disease, peptic ulcer disease (PUD), mild liver disease (LiverMild), diabetes mellitus (DM), DM with chronic complications (DMcx), paralysis, renal disease, cancer, severe liver disease (LiverSevere), metastasis and HIV. Darker shade represents higher co-occurrence.

A total of 77 524 multimorbid patients were seen in five stacked oblong clusters, when first two principal dimensions were plotted (Figure 2A). This contained 72.96% patients in cluster 1, 17.27% patients in cluster 2, 5.25% patients in cluster 3, 4.01% patients in cluster 4 and 0.51% patients in cluster 5 (Figure 1B). Principal dimensions dictating the clustering of patients were seen to be dependent on existence of paralysis, stroke and dementia in patients (Figure 2B). Figure 3 shows basic demographic features underlying each of the disease clusters. A decreasing trend of the proportion of females was noted as we move from cluster 1 to 5 (Figure 3A). The life expectancy shows an increasing trend from cluster 1 to 5 (Figure 3B). Patients in cluster 2 to 5 are functionally not much active due to high number of stroke and paralysis. Cluster 5 has the highest life expectancy with patients having both paralysis and stroke and therefore might be bed-ridden leading to poor quality of life. Age is a major risk factor for dementia [31], and highest number of dementia cases was noted in cluster 5 (Figure 4A) which had the highest life expectancy (Figure 3B). An increasing trend towards multimorbidity signified by increase in CCI was noted as we move from cluster 1 to cluster 5 (Figure 3C). Figure 3D shows the Index of Multiple Deprivation (IMD) as formalized by England for the patients in each cluster. The IMD scores for the first three clusters are similar, followed by an increasing trend, with the highest IMD for cluster 5.

Figure 4A shows a relatively smaller prevalence of diseases in cluster 1, which gradually increases in number of conditions as we move towards cluster 5. Out of all the Charlson’s broad disease classes, pulmonary disease have the maximum prevalence in cluster 1, as 49.32% of patients have pulmonary disease, followed by diabetes mellitus (33.01%) and cancer (32.04%). Cluster 2 is majorly dominated by stroke (77.18%) followed by pulmonary disease (38.59%), diabetes mellitus (26.62%) and cancer (25.20%). Cluster 3 shows the dominance of 73.27% stroke, followed by dementia (35.55%) and paralysis (27.20%). Cluster 4 is dominated by paralysis (99.26%), followed by stroke (98.78%) and pulmonary disease (27.76%). However, dementia in this cluster is negligible (only 2.31%). Finally, all the cluster 5 patients have both paralysis and stroke. Dementia is also one of the major diseases with 85.8% of prevalence in cluster 5. Figure 4B shows a co-occurrence matrix for disease classes in each of the identified disease clusters. Cluster 4 has higher cases of patients having both stroke and paralysis, whereas cluster 5 showed co-occurrence of stroke, paralysis and dementia (Figure 4B). Since clusters 1 to 5 were majorly defined by paralysis, stroke and dementia, we went on first visually inspecting their presence in each cluster (Supplementary Figure 2). This was followed by investigation of the prevalence and co-occurrence of their subclasses (Supplementary Figure 3). Hemiplegia (G81) in paralysis, cerebral infraction (I63), other cerebrovascular disease (I67), sequelae of cerebrovascular disease (I69) in stroke, vascular dementia (F01), unspecified dementia (F03) and delirium (F05) in dementia were found to be more prevalent (Supplementary Figure 3).

### Disease–disease interaction network

**Figure 5:**
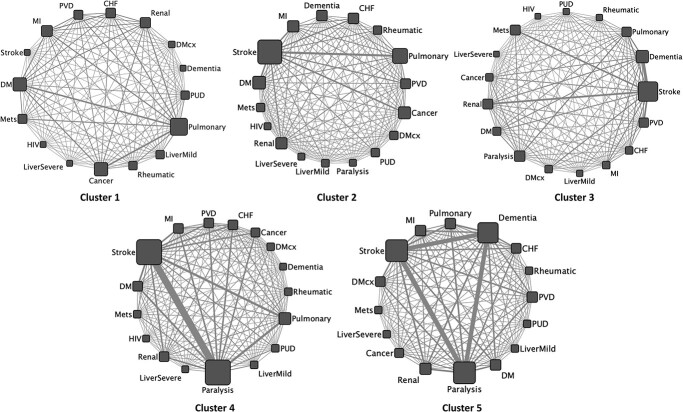
Disease–disease interaction network of 17 broad disease classes as per Charlson’s comorbidity classification. Node size is proportional to the disease prevalence and edge thickness is proportional to the disease co-occurrence. CHF = congestive heart failure, DM = diabetes mellitus, DMcx = DM with chronic complications, Mets = metastasis, MI = myocardial infraction, PUD = peptic ulcer disease, PVD = peripheral vascular disease.

Figure 5 shows a disease–disease interaction network that was obtained for each of the five identified disease endotypes of patients with multimorbidity. Cluster 1 clearly shows dominance of pulmonary disease (largest node) and its strong interaction (thick edges) with diabetes mellitus, cancer, renal disease, peripheral vascular disease, congestive heart failure and myocardial infraction. Unlike other clusters, in cluster 1 there is smaller prevalence of multiple diseases and general interaction (co-occurrence) between them. Cluster 2 is majorly containing the patients with stroke and showing strong interaction with pulmonary disease, diabetes mellitus and cancer. Like cluster 2, cluster 3 is also dominated by stroke, but unlike cluster 2 a strong interaction between stroke and dementia can be easily seen in cluster 3. In general, interaction pattern of cluster 3 is very different from cluster 2 although both are driven by stroke. Cluster 4 and 5 show strong prevalence and interaction between paralysis and stroke. Finally, the cluster 5 has a prominent triad of paralysis, stroke and dementia, showing their strong prevalence and interaction.

## Discussion

Research in the healthcare sector is mostly focused on individual long-term conditions in a structured and standardized way. The traditional approach of treating each disease individually put the patients with multimorbidity under multiple treatments and often this brings its own issues as multiple medications (polypharmacy) can conflict and cause unwanted side effects. Furthermore, patients with multimorbidity are often excluded from clinical trials. As a result, medicines are developed and tested with a single disease focus. There has been recent consensus amongst clinicians and researchers that this trend may not be appropriate for a patient with multimorbidity. The elderly are the highest consumers of prescribed medications and over 50% of whom suffer from multimorbidity. This results in higher medication and undesirable sequelae. Further, patients have different demography and diverse genetic makeup. Prescribing the same treatment to everyone adds to the burden of higher medication and poor drug compliance. This is leading to an increased dissonance between the existing healthcare regimes and the need for the patients they serve. Healthcare should be more holistic and person-centred and hence there is a need to understand multimorbidity better and explore sophisticated, personalized diagnosis and treatments for the same. Otherwise, in future multimorbidity will become more challenging for clinicians, patients and system.

Multimorbidity needs to be managed more efficiently by general practitioners (GPs) or geriatricians in the primary care setting, as the specialists in secondary care tend to focus on only one index disease condition. Managing multimorbidity is tricky as there are so many diseases which require treatment together. The effectiveness of treating patients with multimorbidity should be assessed not just by disease specific indices but by indices such as quality of life, which includes not only symptoms and physical function but also mental health and longevity. National Institute for Health and Care Excellence (NICE) guidelines [32] have also provided suggestions for clinical assessments and management, wherein they are suggesting tailoring the approach to care. Further, they have also provided guidelines to assess the frailty of the patients with multimorbidity. Although NICE guideline of tailoring care for multimorbid patient exists [32], there is little guidance available for managing patients with multimorbidity. This calls for the need to develop efficient and effective strategies for screening and stratifying patients with multimorbidity. Implementation of the analytical approach (MulMorPip) developed in our study suggests five endotypes of multimorbidity which can aid GPs in prioritizing treatment and better management of patients with multimorbidity.

A robust individual becomes frail with age, leading to multimorbidity, which can further lead to disability. Although this simplification is generally true, they (frailty, multimorbidity and disability) may exist independently as well as have some intersections [33]. Multimorbidity may modify the health outcomes and lead to an increase disability or a decreased quality of life or frailty [34]. Cluster 1 patients do not have paralysis and very few have stroke, whereas all cluster 5 patients have both paralysis and stroke (Supplementary Figure 2), suggesting functional impairment to be a major cluster driving feature. An increasing trend in the cases of dementia (Figure 4A) was noted along with the increase in the life expectancy (Figure 3B). A high degree of multimorbidity in dementia patients was noted in Cluster 5 (Figures 4B and5), which was the oldest cohort. Recent research [35] shows the need for identifying modifiable risk factors and pathways common to multimorbidity that can aid in delaying the age-dependent deterioration in patients. The proportion of female participants in cluster 1 is highest (45%) compared to other four clusters where generally a decreasing trend was noted (Figure 3A). This is in line with the literature [36], which suggested that females have relatively lower risk for multimorbidity as the CCI increases from cluster 1 to cluster 5 (Figure 3C). Further the previous studies [36, 37] also suggest that people with low socioeconomic status are more likely to develop multimorbidity, which we confirmed with IMD - England (Figure 3D). This is because socioeconomic status is often related to eating habits and lifestyle [38].

An interesting pattern can be seen in cluster 3. Patients in this cluster majorly either have paralysis or both stroke and dementia (Supplementary Figure 2). Contrary to cluster 4 and 5, cluster 3 patients never had stroke and paralysis together. In fact, stroke patients in cluster 3 had dementia as the major comorbidity (Figure 5). While validating the clusters, the minimum Jaccard similarity of 60% was seen for cluster 3 (Figure 1B). Upon further investigation, we found that most of them were getting misclassified as cluster 2, probably due to a similar comorbidity pattern seen for cluster 2 and 3 (Figure 4B). However, stroke’s comorbidity with dementia can be seen as the major differentiating factor between the two. We confirmed the same by extracting all the cluster 3’s misclassified 305 patients as cluster 2 (Supplementary Table 3). They all were found to be having no dementia. Stroke can lead to dementia, specifically vascular dementia [39]. Since dementia was present in all five clusters, we investigated the prevalence and co-occurrence of subtypes of dementia (Supplementary Figure 3). Cluster 1 predominantly contained delirium (F05) and did not show any preferential comorbidity pattern with any other diseases. Cluster 2 and 3 which were predominated by stroke subtypes—cerebral infraction (I63) and other cerebral vascular disorders (I67)—showed different preferences in terms of their comorbidity pattern with dementia subtypes. While cluster 2 dementia subtypes did not show any preferential comorbidity pattern with any other diseases, cluster 3 dementia subtypes were mostly partnering with I63 and I67. Cluster 4 and 5 were predominated by stroke subtypes—I63, I67 and sequelae cerebrovascular disease (I69). However, while cluster 4 dementia subtypes did not show any preferential comorbidity pattern with any other diseases, cluster 5 dementia subtypes were mostly partnering with I63, I67 and I69.

Stroke most often leads to paralysis [40]. Further, the location of injury dictates the type of paralysis. A spinal cord stroke can lead to tetraplegia (quadriplegia) or paraplegia (ICD-10: G82), whereas a brain injury can lead to hemiplegia (ICD10: G81), i.e. left-side or right-side paralysis for right or left hemisphere injury [40]. Hemiplegia (G81) was the most prevalent type of paralysis in both clusters 4 and 5, and it was noted to be comorbid with both stroke and dementia in cluster 5, whereas in cluster 4 it was noted to be comorbid with only stroke (Supplementary Figure 3B).

Multimorbidity involves a complex interaction between genetics, biobehavioural and socioenvironmental factors. Further, the absence of disease is linked to the balance of proinflammatory and anti-inflammatory activities that can vary across the time course [41]. For patients with multimorbidity, multiple chronic conditions often interact with each other. Thus, finding such interactions and/or associations can contribute to the integrative healthcare approach for the patients with multimorbidity. We have characterized a disease–disease interaction network (Figure 5) for each of the five subgroups or endotypes of patients with multimorbidity. These networks were dominated by interaction between stoke, paralysis and dementia.

A recent study [42] worked on investigating the heterogeneity of diabetes and showed seven distinct clusters of the disease using only six variables. Such stratification of patients can help clinicians to better understand the disease subtypes, their progression and interaction with other diseases, and eventually inform a more personalized treatment pathway for each subtype. Our analytical approach (MulMorPip) is a step towards stratifying multimorbid patients into five endotypes using a very unbiased dataset of UK Biobank. We were further able to validate these endotypes of disease clusters using ML techniques. These endotypes may be considered by a specialist in secondary care, to stratify patients more efficiently for various treatments. For example, a paralysis specialist may want to classify their multimorbid patients into clusters 2 to 5 (Figure 4). These endotypes of patients might be at different stages of their disease progression and/or respond differently to different drugs as they are fundamentally different in terms of disease–disease interaction (Figure 5). Further, we also speculate that these endotypes might be related to risk of early onset and prognosis of certain diseases and thus can be helpful in stratification and prioritizing treatment for the high-risk patients. In terms of future directions, further research is needed to investigate the onset of diseases, their progression and treatment response in these endotypes.

Our study on multimorbidity is limited to the analysis of selected variables, namely disease diagnosis, gender, age and IMD. Future studies can extend on genomic, imaging, biochemical and other datasets present in the UK Biobank. Also, another limitation is that the UK Biobank cohort is heavily dominated by white ethnicity. Therefore, our results may not be generalisable to other ethnicities such as Asian, African or mixed, and thus would require independent validation studies in these cohorts. While our analytical approach (MulMorPip) shows a strong promise of a specific clinical application of patient stratification problem in the field of personalized medicine, it can be adapted and improvized for much wider applications in the field of bioinformatics.

## Authors’ Contributions

P.S. and A.J.B. conceived the project and helped in data interpretation, reviewing and editing of the manuscript. B.P. performed data analysis, data visualization, data interpretation, built the analysis pipeline MulMorPip and wrote first draft of the manuscript.

**Key Points**

- Stratified or personalized medicine and multimorbidity is becoming one of the priority research areas for Innovate UK, the Medical Research Council (MRC) UK and the Academy of Medical Sciences UK.
- In this study, we have developed multimorbidity analysis pipeline (MulMorPip) for stratifying patients with multimorbidity.
- We have then implemented this pipeline to stratify UK Biobank cohort which is a rich biomedical database of approx. 500 000 patients, and have identified and characterized five endotypes of multimorbidity.
- We have also validated our findings (endotypes) using machine learning and were able to classify 20% test set patients with an accuracy of 97% and an average Jaccard similarity of 84%.
- Identified five endotypes draw attention to dementia, stroke and paralysis as important drivers of multimorbidity stratification.
- Inclusion of such patient stratification at the primary care setting can help the general practitioners to better observe the patients’ multiple chronic conditions, their risk stratification and personalization of treatment strategies.

## Supplementary Material

MulMorPip_Supplementary_Data_v15_bbac410

## Notes

**Bodhayan Prasad** is a PhD Researcher at the Personalised Medicine Centre, School of Medicine, Ulster University, UK. He holds a MSc in Computational and Integrative Sciences from Jawaharlal Nehru University, India.

**Anthony J. Bjourson** is an Emeritus Professor of Genomics at the Personalised Medicine Centre, School of Medicine, Ulster University, UK. He holds a PhD in Genomics and Molecular Biology from Queen's University, Northern Ireland.

**Priyank Shukla** is a Lecturer in Stratified Medicine (Bioinformatics) at the Personalised Medicine Centre, School of Medicine, Ulster University, UK. He holds a PhD in Computer Science with area of research in Bioinformatics from University of Bologna, Italy.
