# Supplementary material for: Data-driven patient stratification of UK Biobank cohort suggests five endotypes of multimorbidity
Source: Brief Bioinform. 2022 Oct 8;23(6):bbac410. doi: 10.1093/bib/bbac410 (PMC9677496; doi:10.1093/bib/bbac410)
Supplement: MulMorPip_Supplementary_Data_v15_bbac410 [file mulmorpip_supplementary_data_v15_bbac410.pdf]

Supplementary Data

Supplementary Table 1. Distribution of number of diseases versus number of patients with multimorbidity in UK Biobank. Multimorbidity implies occurrence of two or more diseases.

No. of diseases	No. of patients
2	44,778
3	18,501
4	8047
5	3647
6	1596
7	675
8	213
9	51
10	15
13	1
**Total**	**77,524**

Supplementary Table 2. Demographics of multimorbid patient's cohort in UK Biobank. Cohort is further presented based on cluster affiliation (rows) as per Figure 2, training-test split (columns) used in cluster validation as per Figure 1B and overall cohort characteristics (last row). P = *P*-value of either 2-sample proportion test or 2-sample t-test, as appropriate.

Clusters	Proportion of Females, n (%)				Age at death, mean (s.d.), years				CCI, mean (s.d.)				IMD – England, mean (s.d.), years			
	Train	Test	Total	P	Train	Test	Total	P	Train	Test	Total	P	Train	Test	Total	P
1	20545 (45.3)	5140 (45.7)	25685 (45.4)	0.51	69.9 (6.9)	70.0 (6.7)	69.9 (6.9)	0.59	4.0 (2.2)	4.0 (2.2)	4.0 (2.2)	0.88	20.8 (15.8)	20.9 (15.7)	20.8 (15.8)	0.67
2	4501 (42.3)	1144 (41.8)	5645 (42.2)	0.64	72.5 (6.4)	72.2 (6.2)	72.2 (6.3)	0.33	4.1 (2.3)	4.1 (2.3)	4.1 (2.3)	0.78	21.0 (15.8)	21.1 (16.2)	21.0 (15.9)	0.77
3	1446 (44.0)	318 (40.7)	1764 (43.3)	0.10	71.9 (6.9)	71.6 (6.8)	71.9 (6.9)	0.43	4.9 (2.5)	4.8 (2.5)	4.9 (2.5)	0.34	20.3 (15.6)	20.9 (16.2)	20.4 (15.7)	0.44
4	1014 (41.1)	271 (42.1)	1285 (41.3)	0.68	70.8 (7.2)	71.5 (7.1)	71.0 (7.2)	0.26	5.5 (2.6)	5.5 (2.5)	5.5 (2.6)	0.64	22.0 (16.3)	21.5 (16.3)	21.9 (16.3)	0.55
5	116 (38.0)	32 (36.0)	148 (37.6)	0.82	74.9 (4.6)	74.3 (4.8)	74.7 (4.6)	0.52	6.8 (2.5)	6.9 (2.3)	6.9 (2.4)	0.72	23.2 (15.6)	22.7 (17.5)	23.1 (16.1)	0.79
Overall	27622 (44.5)	6905 (44.5)	34527 (44.5)	1.00	70.7 (6.9)	70.7 (6.7)	70.7 (6.9)	0.95	4.2 (2.3)	4.2 (2.2)	4.2 (2.3)	0.87	20.9 (15.8)	21.0 (15.8)	20.9 (15.8)	0.59

Supplementary Table 3. Confusion matrix for cluster validation on 20% model-blind test set. Each cell represents number of patients.

		**Predicted**				
	**Cluster No.**	**1**	**2**	**3**	**4**	**5**
**Actual**	**1**	11235	14	0	0	0
	**2**	97	2639	4	0	0
	**3**	0	305	473	4	0
	**4**	0	10	5	625	4
	**5**	0	0	0	15	74

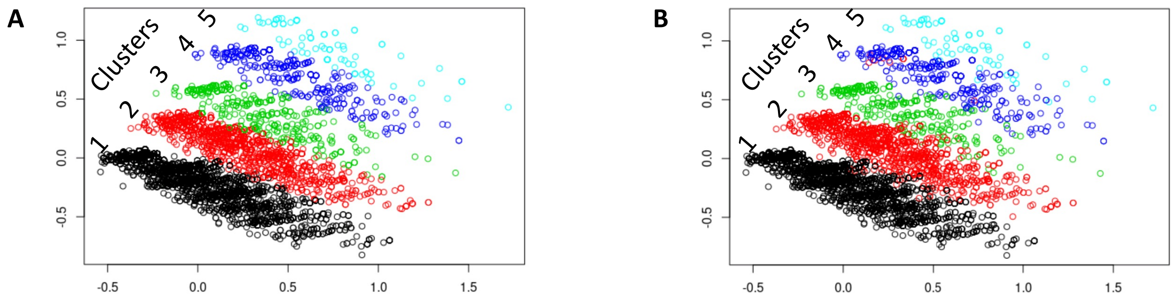

Supplementary Figure 1. (**A**) The MCA plot of 20% test set, coloured by the original cluster numbers. (**B**) The MCA plot of 20% test set, coloured by the cluster numbers obtained from the DTC model. MCA = multiple correspondence analysis and DTC = decision tree classifier.

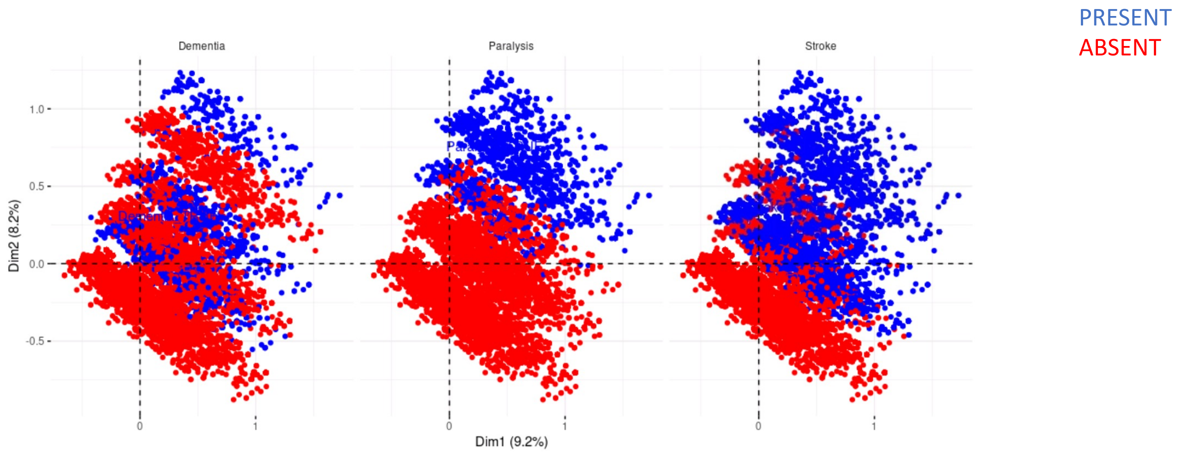

Supplementary Figure 2. MCA plot showing five different multimorbid clusters labelled for the presence or absence of dementia, stroke and paralysis. MCA = multiple correspondence analysis.

A

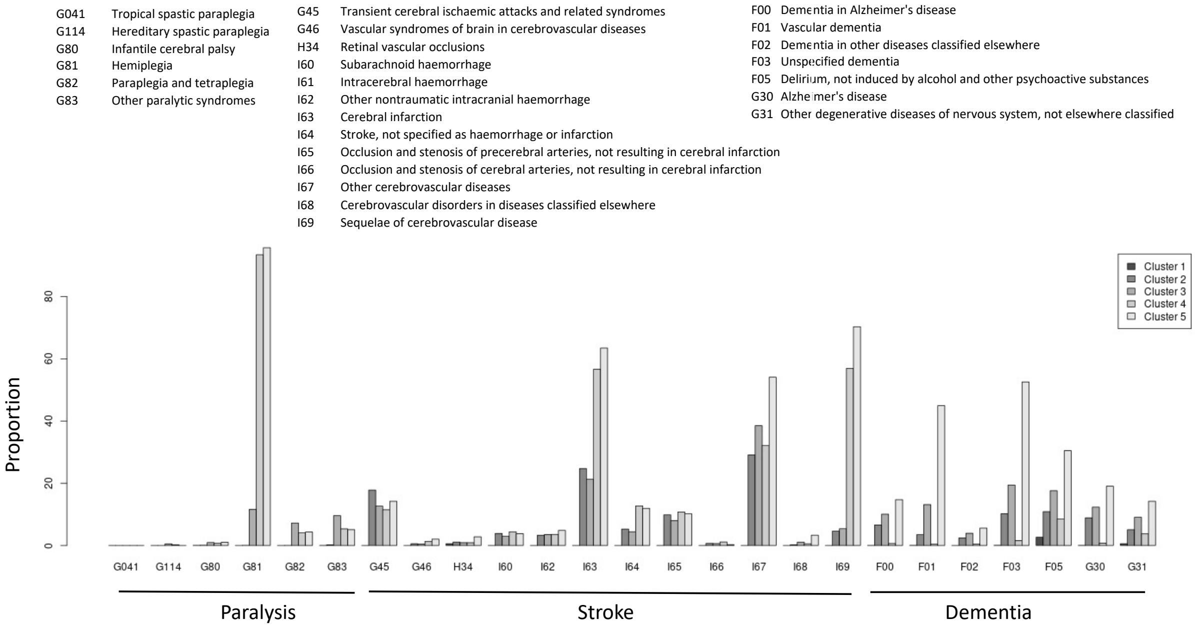

B

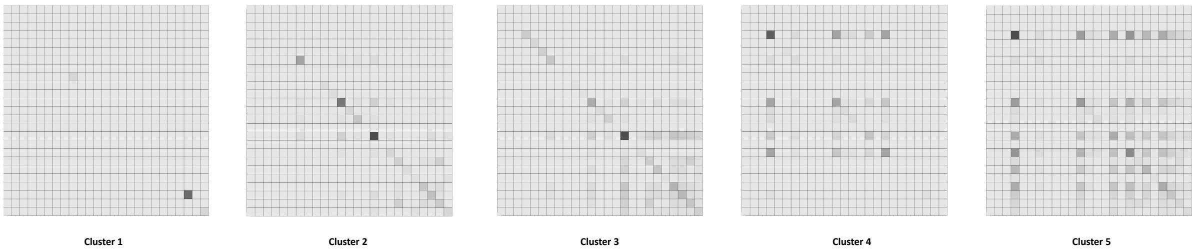

Supplementary Figure 3. (**A**) Prevalence of subclasses of paralysis, stroke and dementia. (**B**) Heatmap showing the co-occurrence of subclasses of paralysis, stroke and dementia. Order of disease from top to bottom and left to right are: G041, G114, G80, G81, G82, G83, G45, G46, H34, I60, I62, I63, I64, I65, I66, I67, I68, I69, F00, F01, F02, F03, F05, G30 and G31. Darker shade represents higher co-occurrence.
